# Masking Emotions: Face Masks Impair How We Read Emotions

**DOI:** 10.3389/fpsyg.2021.669432

**Published:** 2021-05-25

**Authors:** Monica Gori, Lucia Schiatti, Maria Bianca Amadeo

**Affiliations:** Unit for Visually Impaired People (U-VIP), Istituto Italiano di Tecnologia, Genova, Italy

**Keywords:** COVID-19, emotion inference, facial configuration, social development, face mask

## Abstract

To date, COVID-19 has spread across the world, changing our way of life and forcing us to wear face masks. This report demonstrates that face masks influence the human ability to infer emotions by observing facial configurations. Specifically, a mask obstructing a face limits the ability of people of all ages to infer emotions expressed by facial features, but the difficulties associated with the mask’s use are significantly pronounced in children aged between 3 and 5 years old. These findings are of essential importance, as they suggest that we live in a time that may potentially affect the development of social and emotion reasoning, and young children’s future social abilities should be monitored to assess the true impact of the use of masks.

## Introduction

Understanding emotions is crucial for social interaction. Specifically, interpreting other people’s facial configurations is fundamental during social development when children learn to interact with others (Denham et al., 2014). The first ability to discriminate facial configurations associated with emotional states develops early in infancy. Children within the first months of life begin to understand positive and negative emotions (Walker-Andrews, 1997; Grossmann, 2010). For example, at 4 months of age, infants begin to discriminate facial movements associated with anger and happiness emotions (Barrera and Maurer, 1981). At a few months, infants can discriminate facial movements expressing surprise from those associated with happiness and sadness, and they can discriminate between different emotion intensities (i.e., mild vs. intense happy facial configuration; see de Haan and Nelson, 1997 for a review of studies). Within the first year of life, infants start to engage in social referencing, i.e., they use the adult caregiver’s facial movements to adjust their social behavior (Hertenstein and Campos, 2004). Within the second year of life, children develop more abstract concepts of emotion, like understanding the congruence of other people’s facial movements and actions (Hepach and Westermann, 2013).

While essential emotion processing is evident in infants, early childhood is considered a critical period for the development of understanding emotions and emotion processing (Denham et al., 2003). Continuous developmental changes do indeed occur from infancy to adulthood, as the individual’s social environment gradually grows in its capacity and complexity (Herba and Phillips, 2004; Tonks et al., 2007; Barrett et al., 2019). Developmental studies of emotion processing in the preschool years have shown that children’s explicit recognition of emotional states emerges along with their development. Happiness is recognized earliest and most accurately, followed by sadness or anger, and then by surprise or fear (Camras and Allison, 1985). Around 4–15 years old children’s accuracy in recognizing sad facial configurations improves with age at a slower rate compared to facial movements expressing happiness, fear, and disgust (Herba et al., 2006). Higher misidentification for sad faces still has been observed in 10-year-old compared to adults (Gao and Maurer, 2009). Children under 11 years make more errors in emotional labeling states expressed by facial configurations than children in early adolescence (Tonks et al., 2007). Moreover, the ability to infer emotions from eye movements and speech, does not stabilize until middle childhood and adolescence (Herba et al., 2006). Finally, research studying the neural substrates associated with observation of different facial movements supports previous results showing that the processes of emotional reasoning are not adult-like until early adolescence (Batty and Taylor, 2006). Thus, these results suggest that the development of emotion reasoning and, in particular, the inference of emotional states from face observation, continues to develop from preschool to middle childhood and adolescence (Herba et al., 2006). With increasing age, emotion inference from facial stereotypes is probably more rapid due to increased efficiency in decoding faces (Chung and Thomson, 1995; De Sonneville et al., 2002).

Since, as we have seen, the most immediate way to read other people’s emotions is through observation of facial movements, an interesting question is how the ability to recognize facial configurations changes when a part of the face is covered. Currently, due to the COVID-19 emergency, we are all facing a natural reduction of accessibility to facial information during interactions. Other people’s faces have to be processed with masks, which obscure the visual information from the mouth and the lower part of the face. Here, we hypothesize that recognition of facial configurations associated with specific emotions should be affected by the mask’s presence. We also hypothesize that this effect should be stronger for younger children for whom the developmental trend for inferring emotions from facial movements is still an ongoing process. Thus, we studied if face reading is more challenging when the mouth is not visible, while wearing a face mask. This issue was recently addressed in the context of the COVID-19 pandemic by Freud et al. (2020), who evaluated how the face masks change the ways in which faces are perceived on a large sample of adults. They provided evidence of a quantitative decrease of face processing abilities in the presence of a face mask and an indication of qualitative changes in face processing (i.e., the process of face features becomes less holistic).

Moreover, a recent study (Ruba and Pollak, 2020) investigated the implications of mask-wearing on inferring emotions from facial configurations using a sample of children aged between 7 and 13 years old. They revealed that children could make above-chance inferences about emotions even when parts of the faces were covered, concluding that masks are unlikely to produce serious impairments of children’s social interactions. Here, we expanded this topic (i) by involving preschoolers aged between 3 and 5 years old, children aged between 6 and 8 years old, and adults and (ii) by including also facial configurations associated with a neutral state and with different intensities of emotions. We expected facial obstruction to be significantly more compromising for younger children who are still developing their emotional reasoning and social interaction skills. Indeed, exposition to faces in infancy and early childhood is essential in developing abilities that are strongly involved in emotional reasoning. For instance, short-term and long-term visual memory influence how children reason about their and others’ emotions. Children develop a bias in labeling facial configurations, such as angry or happy, based on familiarity, i.e., the frequency at which they have been exposed to similar facial configurations in their environment. In this vein, they tend to label faces as happy in the first years of life, since they are less exposed to angry or sad faces, while children with a history of abuse more frequently tend to label faces as angry (Malatesta et al., 1989; Pollak and Kistler, 2002).

The current crisis presented us with a unique occasion to study how face reading changes during development in children and within adults, while both are forced to interact with people wearing face masks. To this end, different age groups’ abilities to make inferences from facial configurations were studied in the context generated by the COVID-19 emergency. We performed our experiments at the beginning of the crisis (within 2 weeks after Italy’s first lockdown phase) when children and adults were exposed to masks for the first time and had to deal with the ability to identify facial movements with the presence of a mask. This condition was a new experience for all the participants, which guaranteed that the performance was not affected by experience or previous exposition.

## Materials and Methods

A group of 119 participants was recruited from the general population: 31 toddlers from 3 to 5 years of age (i.e., preschool age, mean ± SD: 4.3 ± 0.7 years old), 49 children from 6 to 8 years of age (i.e., school age, 6.8 ± 0.8 years old), and 39 adults from 18 to 30 years of age (27.4 ± 2.1 years old). Subjects were native Italian speakers. The ethics committee approved the research protocol of the local health service (Comitato Etico, ASL3 Genovese, Italy) and informed consent was obtained before submitting the questionnaire.

Different methods exist to assess the development of emotion reasoning, i.e., the ability to use expressive behavior and contextual information to make inferences and predictions about other people’s emotional states and actions (Ruba and Pollak, 2020), including non-behavioral (functional and structural MRI and electroencephalography) and behavioral approaches (Paiva-Silva et al., 2016). The latter is the most widespread, and typically consists of static (images) or dynamic (brief videos) human face stimuli. Such tests have been widely used in different domains to assess level of impairment in recognition of facial movements in the presence of psychiatric (Rocca et al., 2009) or movement (Argaud et al., 2018) disorders, autism (Wieckowski et al., 2020), and developmental disabilities (Rojahn et al., 1995). It is essential to carefully select the task characteristics since they have been proven to influence performance (Hayes et al., 2020). Typically, most research in the field relied on paradigms that require labeling of static facial configurations, which are often stereotypes, i.e., posed configurations not authentically expressing the targeted emotion. Ruba and Pollak (2020) documented the limitations of such methods for measuring emotions in that they: (1) focus on the face only, ignoring the impact of contextual information in making emotion judgments, (2) use static and posed configurations that may not generalize to how we infer emotions in everyday life, and (3) examine a limited range of emotions. Being aware of the meaning and constraints of such a paradigm, in the present study, we chose a standardized verbal-response test based on selecting an emotion’s label (forced-choice) to describe static pictures of human facial configurations. Such choice was taken to ensure repeatability of the task and to make the administration of the test easy for the subjects *via* smartphone to overcome the difficulties related to social distancing rules. To partially overcome the intrinsic limits of such test and capture a wider variation in facial movements expressing emotions, we considered facial configurations expressing two levels of intensity associated with the same emotion. All participants completed an internet-based questionnaire shown on a smartphone that required them to identify facial emotions on static pictures with and without facial masks. To control for face mask exposure, the test was performed between 5 and 15 days after the first lockdown ended in Italy (May 2020). The task was structured in sequential blocks, showing firstly a set of pictures with facial masks, followed by a block of mask-free images. A total of 40 adult face pictures were presented in randomized order including four repetitions of four facial emotions (happiness, sadness, fear, and anger) with two levels of intensity (mild, extreme), and a neutral facial expression that was presented for eight times to each participant. Both the original and modified pictures were obtained from the ER-40 color emotional stimuli database (Gur et al., 2002; Pinkham et al., 2008), developed for the validated ER-40 test for facial emotion recognition (Kohler et al., 2004; Carter et al., 2009). For the set of images containing masks, pictures from the original database were modified *ad hoc* by a web designer who created and added realistic face masks. Participants were asked to identify the facial emotion by choosing five possible randomized options: happy, sad, fearful, angry, and neutral. Toddlers were guided in the task by a caregiver (typically a parent). Although, we could not ensure complete control over test administration, caregivers were provided with specific written instructions about their role. The caregiver had to read the question to the child, while showing the current image, read the response options, and select the child’s choice. Caregivers were carefully instructed not to influence, in any way, the answers of the child. No time limits were imposed to provide an answer.

For data analysis, performance was calculated as a percentage of correct responses with and without the mask. From this, the impairment due to the mask’s presence was calculated as the difference between the percentage of correct responses with and without the masks. First of all, *t*-tests were conducted to statistically compare the performance in each condition (Mask, NoMask) and age group (i.e., Toddlers, Children, and Adults) to chance level responding (i.e., 20%). Results were corrected for multiple comparisons using Bonferroni correction. Subsequently, performance was analyzed with a two-way ANOVA considering conditions (i.e., Mask, NoMask) as within-subject factor and groups (i.e., Toddlers, Children, and Adults) as between-subject factor. To investigate possible differences associated with intensities of emotions or neutral expressions, the intensity of emotions (i.e., Low, High) and presence of emotional content (i.e., Emotion, Neutral) were also separately considered as independent variables in the ANOVA on performance. Besides this, one-way ANOVA with impairment as dependent variable and group (i.e., Toddlers, Children, and Adults) as a between-subject factor was also performed. *Post hoc t*-tests were carried out applying Bonferroni correction to results.

## Results

The main insight of the present research is that face masks’ use influences emotion inference from faces for all ages and especially for toddlers.

**Figure 1:**
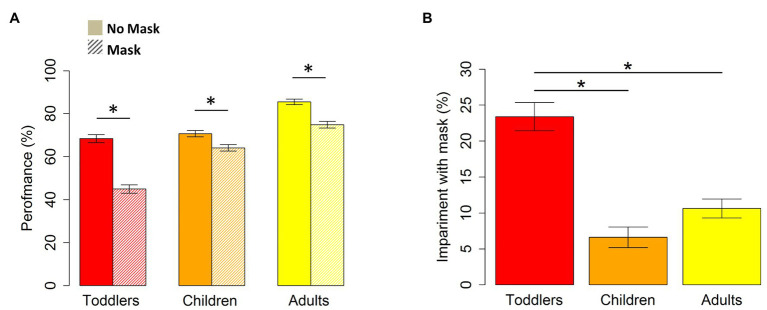
**(A)** Percentage of correct responses without and with the masks in toddlers, children, and adults. **(B)** Percentage of impairment due to masks in toddlers, children, and adults. Impairment is calculated as the difference between the percentage of correct responses without and with the masks. The SEM is reported. The stars indicate a significant difference between the groups (*p* < 0.001).

The ability of inferring emotions from facial configurations was, for all participants, significantly above-chance level both without (for toddlers: t_30_ = 21.83, *p* < 0.001; for children: t_48_ = 35.68, *p* < 0.001; for adults: t_38_ = 71.63, *p* < 0.001) and with (for toddlers: t_30_ = 8.29, *p* < 0.001; for children: t_48_ = 25.96, *p* < 0.001; for adults: t_38_ = 40.1, *p* < 0.001) face masks. However, the two-way ANOVA with performance as dependent variable and condition (i.e., Mask, NoMask) and group (i.e., Toddlers, Children, and Adults) as independent variables revealed a main effect of condition (F_1,116_ = 48.7, *p* < 0.001, ges = 0.4), a main effect of group (F_1,116_ = 190.2, *p* < 0.001, ges = 0.2), and a significant interaction between them (F_2,116_ = 29, *p* < 0.001, ges = 0.1). Thus, for all groups, the percentage of correct responses is significantly reduced for the images with face masks compared to the images without face masks (for toddlers: t_30_ = 11.94, *p* < 0.001; for children: t_48_ = 4.61, *p* < 0.001; for adults: t_38_ = 8.1, *p* < 0.001; see Figure 1A). Moreover, in line with the literature (Chronaki et al., 2015), *post hoc t*-tests replicated the developmental trend showing that toddlers and children are significantly weaker at labeling emotions without masks compared to adults (Figure 1A; for toddlers vs. adults: t_40.2_ = 7.1, *p* < 0.001; for children vs. adults: t_78.9_ = 8.8, *p* < 0.001). However, although toddlers and children have similar performances when no mask is worn (t_54_ = −0.88, *p* = 0.9), the performance of toddlers is more affected by the use of a mask than the performance of both older children (t_49_ = −5.52, *p* < 0.001) and adults (t_42.2_ = 9,02, *p* < 0.001). Also, older children show a lower performance in labeling emotions on images with face masks compared to adults (t_85.2_ = 4.9, *p* < 0.001).

**Figure 2:**
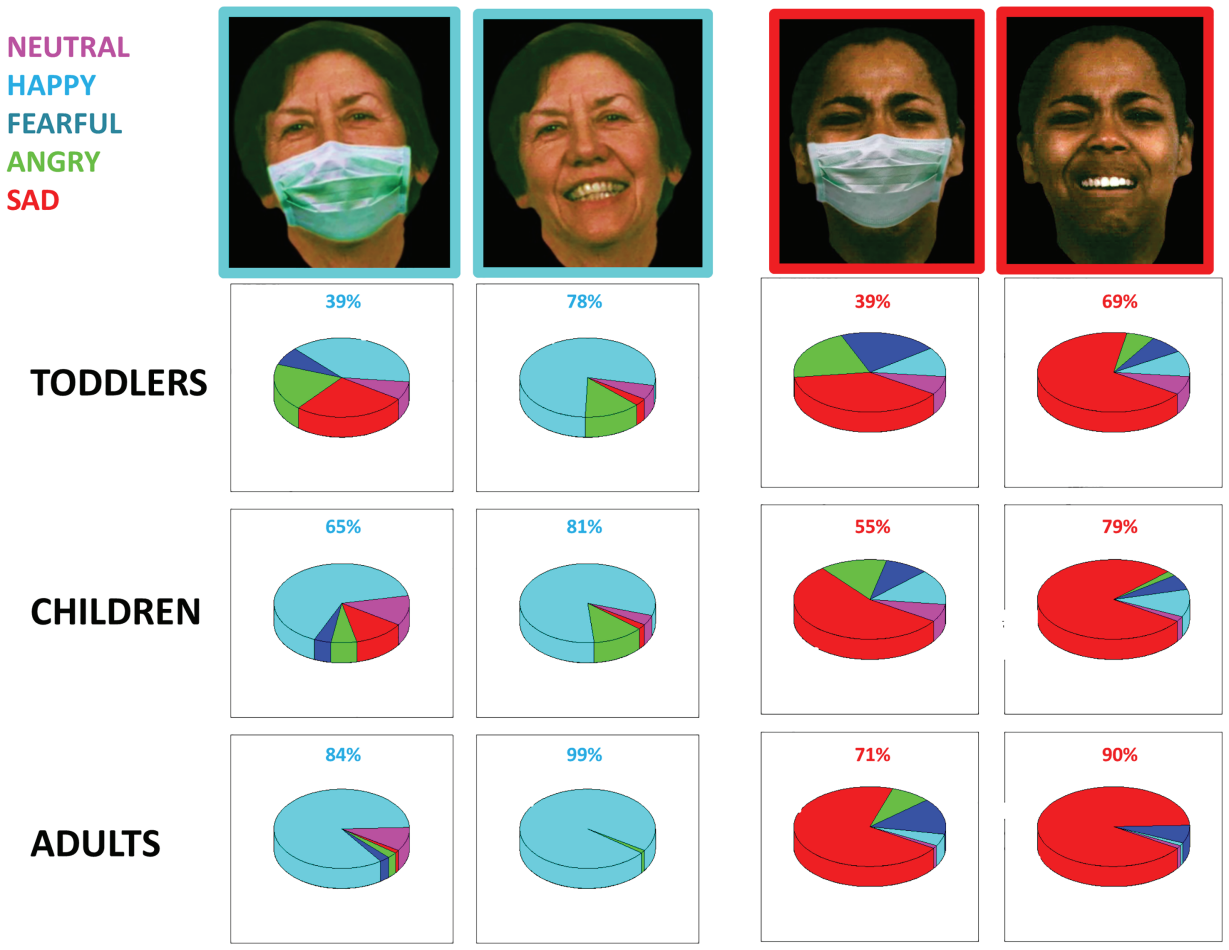
Responses given by toddlers, children, and adults for two exemplar emotions with and without mask: happiness (left) and sadness (right). Percentage of the answer is reported for “Sad” (red), “Happy” (cyan), “Fearful” (blue), “Angry” (green), and “Neutral” (violet). Face images have been obtained from the ER-40 color emotional stimuli public database (Gur et al., 2002; Pinkham et al., 2008).

Responses from toddlers, children, and adults for two exemplar emotions (i.e., happiness and sadness) are reported in Figure 2. The percentage of impairment due to the presence of masks across all groups is displayed in Figure 1B. As shown, participants, and especially toddlers, confuse the correct emotion with other emotions more frequently when the mask is present. The one-way ANOVA confirms this result of impairment due to masks’ presence (F_1,116_ = 28.96, *p* < 0.001, ges = 0.3). The negative effect of the mask is more significant for toddlers aged between 3 and 5 years old compared to older children (t_60.1_ = 6.89, *p* < 0.001) and adults (t_54.4_ = −5.4, *p* < 0.001). Interestingly, no difference is observed between the older children and adults (t_85.9_ = −2.1, *p* = 0.1).

**Figure 3:**
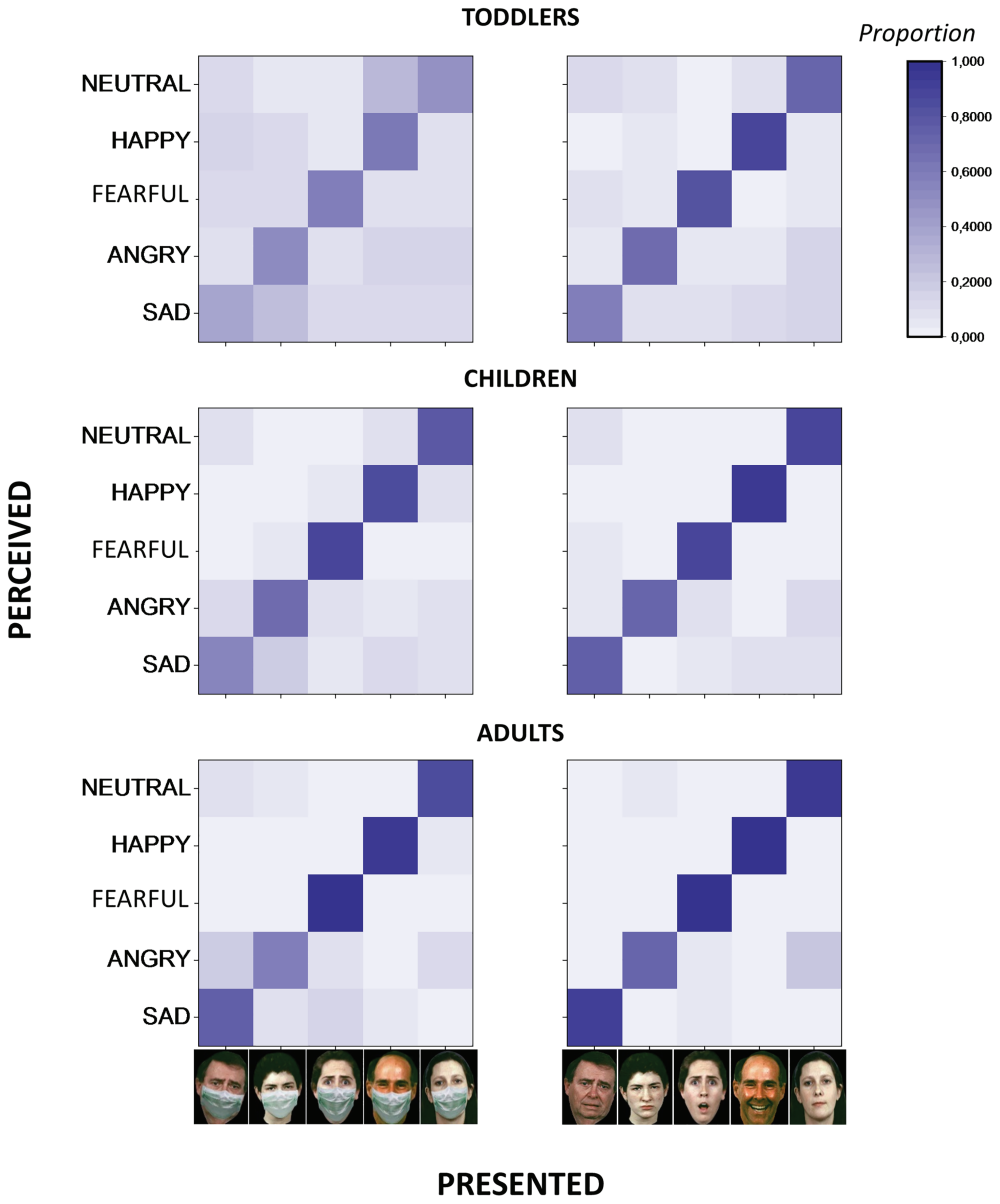
Confusion matrices for emotion inference from facial configurations with (left) and without (right) face masks for the three age groups. On the *x*-axis, presented stimuli. On the *y*-axis, emotion perceived by participants. The proportion of responses is reported as a color map. Face images have been obtained from the ER-40 color emotional stimuli public database (Gur et al., 2002; Pinkham et al., 2008).

Since no interaction between condition (i.e., Mask, NoMask), group (i.e., Toddlers, Children, and Adults) and either intensity (i.e., Low, High; F_2,116_ = 2.06, *p* = 0.1, ges = 0.005) or presence of emotional content (i.e., Emotion, Neutral; F_2,116_ = 1.86, *p* = 0.1, ges = 0.004) was found, the data presented in the previous analyses were merged for these dimensions. Response distribution among different emotions for the different age groups, with and without masks, is represented in Figure 3, which reports the matrices of confusion. Both with and without masks, toddlers and children confuse the correct expression with other expressions more than adults. For all groups, the confusion increases with masks, and this is especially true for toddlers.

## Discussion

### Emotion Inference Without Masks

Identification of emotions and, particularly, of facial movements is fundamental for our ability to interact with others and adjust one’s social behavior accordingly (Philippot and Feldman, 1990; Vicari et al., 2000; Barrett et al., 2019). A consistent body of research mostly focused on preschool-aged children, reported that an inference of emotional expressions gleaned from facial configurations improves during development (Odom and Lemond, 1972; Philippot and Feldman, 1990; Gross and Ballif, 1991; Boyatzis et al., 1993). Changes between children and adults have been described from a neurophysiological perspective (Thomas et al., 2001) and have been related to the development of higher cognitive skills associated with increased efficiency of the pre-frontal neural structures. In line with the literature (Chronaki et al., 2015), we confirmed that toddlers and children are significantly less reliable than adults at labeling emotions from facial configurations without masks, even though their performance is always above chance level.

### Emotion Inference With and Without Face Masks During the Development Stage

This research’s central insight is that there are developmental differences across toddlers, older children, and adults, when inferring emotions from facial configurations and when face masks partially cover faces. Indeed, face masks affect emotion understanding for all ages, but the effect is especially pronounced for toddlers. Although still above chance level, we observed that toddlers’ performance is more influenced by a mask than older children and adults’ performance. All participants, independent of age, and despite variation of facial expression and intensity of emotion, faced more difficulties recognizing emotions with face masks. However, in line with a previous study on the topic (Ruba and Pollak, 2020), children and adults showed a high performance even for images with face masks (i.e., percentage of correct responses higher than 60%). This result suggests that it was still quite easy for them to infer emotions of others with face masks present. On the contrary, the mask’s presence was significantly more impactful for toddlers, causing a higher drop in their performance. This difference could be explained by different age-related developmental stages of face processing associated with emotion reasoning, which is very much still ongoing in early childhood.

### The Use of Facial and Situational Cues to Infer Emotions During the Development Stage

Speaking about the effect that masks can have in real life, we should consider that facial movements and situational cues are crucial when interacting with others. It has been shown that younger children rely on facial expressions to infer information on another’s emotional state to a greater extent than situational cues (Hoffner and Badzinski, 1989). Indeed, the child’s ability to rely on situational cues only increases with age. Around 3–5 years old toddlers focus almost exclusively on facial expressions, whereas children rely on situational cues by 8 or 9 years of age. This evidence could explain the lower performance we observed in younger children when the mask is present. Likely, while adults and older children can use other contextual cues to infer the social content (Leitzke and Pollak, 2016), this may be more difficult for toddlers. Supporting the hypothesis of age-related differences in emotion reasoning, with increasing age, children become more insightful into their own emotional lives and demonstrate an increased understanding of others’ mixed emotions (Campbell et al., 1995; Izard and Harris, 1995; Want et al., 2003). Furthermore, it is known that emotion perception in infancy makes use of social referencing, i.e., infants exploit the caregiver’s facial movements to infer emotional information and direct their behavior in social contexts (Saarni et al., 2007).

These findings, taken together, could explain our observation that toddlers are more affected by the mask’s use, suggesting the importance of facial cues for the early development of emotion reasoning and, likely, social skills. The problem might be overcome in older children, who present more advanced competences in combining facial expressions with situational cues and can therefore extract richer information useful to interact with others, even in the presence of face masks.

### Use of Masks and Social Exposure Reduction

Sensitivity to facial emotion movements is fundamental to children’s emotional processing and social competence. For instance, some recent works showed evidence that our categories for emotions are not fixed and they strongly depend on the types and frequencies of facial movements to which we are exposed (Plate et al., 2019). Therefore, in a critical period regarding the development of emotional categories, young children are likely to be affected by exposure to face masks being worn. It has also been shown that children can better understand facial emotional cues from positive interpersonal relationships over time (Denham, 1998). Also, psychosocial deprivation during critical developmental periods leads to short‐ and long-term consequences, including perturbations at molecular and neuronal levels, as well as psychological and behavioral impairments (Nelson et al., 2019).

It is not clear whether the changes in face configurations’ exposure due to masks’ use might affect the development of emotions’ inference and the development of social interactions’ capabilities in toddlers whose growth was developing at the time of the COVID-19. However, such questions have already received attention at a social and institutional level. In the document drafted by WHO and UNICEF to provide a guide to decision-makers and authorities in public and professional settings on the use of masks for children in the context of the COVID-19 pandemic (World Health Organization, 2020), it is generally the use of masks is generally discouraged when dealing with children up to 5 years old, given that at that age, they achieve significant developmental milestones (Coppola, 2014). For older children, it is advised to carefully weigh the benefits of wearing masks against potential harm, including social and communication concerns. The same considerations hold about adopting a risk-based approach in evaluating the potential impact of mask wearing in social and school settings on children’s learning and psychosocial development. Our study contributes to collect essential information about these aspects scientifically, contributing to defining indicators that help monitoring and evaluating the impact of and exposure to masks’ use on children, with specific attention to emotional reasoning and related developmental factors. Indeed, even though young children are generally exempted from masks’ use, they are still exposed to face masks in different everyday social and educational settings.

Future longitudinal works are necessary to investigate if the social challenge these children are experiencing will impact their future ability to interact with others. Research should also include more informative and generalizable methods to assess emotion reasoning beyond labeling facial configurations. This exploration will be even more important if the use of masks is extended over time. Notably, we performed our experiments at the beginning of the crisis (within 2 weeks after the first lockdown phase). In this period, the face perception with masks was a new experience for all the participants, and the performance was not affected by experience or previous exposition. We plan to replicate this study, i.e., after 1 year of exposition with face-masks. It is indeed possible that toddlers, children, and adults have improved the ability to recognize emotions from facial expressions with masks (e.g., through eyes) due to experience. It would also be interesting to adopt a multimodal approach to test abilities of emotion inference, such as exploring how visual and audio expression information are combined when the mask is used at different ages. It has been shown that vocal and visual information can cooperate to detect expression with face masks during development stages (Chladkova et al., 2021). If a cross-modal association between vocal expressions and eye cues can be used, then it is possible that the vocal cue can be used to train face processing when the mask is present.

### Emotion Reasoning and Disorders

Understanding the effect of long-term reduced facial expression exposure due to face masks being worn might be more critical for children with different impairments and disorders. It has been shown that abnormalities in processing certain emotions may correspond to particular disorders’ symptoms (Phillips et al., 2003a,b). Deficits in emotional reasoning and the ability to infer emotional content from facial configurations have been well demonstrated in children with autism (Hobson et al., 1988, 1989; Celani et al., 1999; Dyck et al., 2001). Moreover, abnormalities in recognition of emotional expressions are associated with psychiatric disorders both in children and adults (Green et al., 2000; Blair, 2003; Phillips et al., 2003b). Also, children with anxiety or depression process emotional information differently than non-anxious or non-depressed children (Ladouceur et al., 2005). Adults (Mogg et al., 2004) and children (Hadwin et al., 2009) with anxiety demonstrate an early processing bias toward anger/threatening expressions, whereas adults with depression take more time before biasing toward expressions of sadness (Gotlib et al., 2004). A greater understanding of the typical development of emotion inference and neural systems associated with this acquisition would facilitate earlier identification and appropriate therapeutic interventions for emerging patterns of aberrant emotional behaviors. Previous studies have already evidenced that visual impairments, specifically age-related macular degeneration, negatively influence social involvement because of a reduced capability to perceive faces (Lane et al., 2018). In the context of the COVID-19 pandemic, it is crucial to understand whether wearing masks can lead to a similar outcome and which categories of people are more at risk in the long term. Particular attention should be devoted to children with disabilities, for whom interacting with people wearing face masks could exacerbate learning and social barriers. For them, the use of adapted transparent masks should at least be explored as an alternative (Sheik-Ali et al., 2021).

## Conclusion

To conclude, here, we showed that mask use influences our ability to infer facial expressions at any age. Furthermore, we showed that the human capacity to read emotions from facial configurations when a face mask is present becomes particularly reduced in toddlers. We suggested that this is related to different age-related developmental stages of face processing associated with emotional reasoning. Such observation poses the question whether a privation of facial visual features, as the one we are experiencing due to the COVID-19 pandemic, might alter or delay the development of social skills associated with face perception in early childhood. Designing devices for personal protection that allows visibility of the lower part of the face may be crucial in all environments important for developing social and interaction skills in children, such as in education or rehabilitation, especially for those suffering from sensory or cognitive deficits. Knowledge from the current study can inform emotion-centered interventions and prevention programs that aim to foster socio-emotional processes linked to emotional understanding (Izard et al., 2008).

## Data Availability Statement

The raw data supporting the conclusions of this article will be made available by the corresponding author on reasonable request. The program code generated during the current study is available from the corresponding author on reasonable request.

## Ethics Statement

The studies involving human participants were reviewed and approved by Comitato Etico, ASL3 Genovese, Italy. Informed consent was obtained from all individual participants included in the study. For minors, written informed consent to participate in this study was provided by the participants’ legal guardian/next of kin.

## Author Contributions

MG and LS contributed to conception and design of the study. MG, LS, and MA collected the data and organized the database. MA performed the statistical analysis. MG wrote the first draft of the manuscript. All authors contributed to the manuscript revision, read, and approved the submitted version.

### Conflict of Interest

The authors declare that the research was conducted in the absence of any commercial or financial relationships that could be construed as a potential conflict of interest.
